# Bromopropylate Imidazoliumyl Substituted Silicon Phthalocyanine for Mitochondria-Targeting, Two-Photon Imaging Guided *in Vitro* Photodynamic Therapy

**DOI:** 10.3389/fphar.2022.921718

**Published:** 2022-07-12

**Authors:** Kuizhi Chen, Jialin Hou, Bingcheng Huang, Shuanghuang Xiao, Xia Li, Hong Sun, Yiru Peng

**Affiliations:** ^1^ Fujian Provincial Key Laboratory of Advanced Materials Oriented Chemical Engineering, Fujian Provincial Key Laboratory of Polymer Materials, College of Chemistry and Materials, Fujian Normal University, Fuzhou, China; ^2^ Department of Breast Surgery, Fujian Medical University Union Hospital, Fuzhou, China; ^3^ Department of Pharmacy, Shengli Clinical Medical College of Fujian Medical University, Fujian Provincial Hospital, Fuzhou, China

**Keywords:** silicon (IV) phthalocyanine, mitochondria-targeting, two-photon fluorescent imaginging, photodynamic therapy, bromopropylate imidazoliumyl

## Abstract

Maximization of phototoxic damage on tumor is essential for effective anticancer photodynamic therapy (PDT). Highly cancer-cell-organelle-specific delivery of efficient photosensitizers (PSs) *in vitro* and *in vivo* is in great demand. In this paper, a novel water-soluble mitochondria targeted cationic bromopropylate imidazoliumyl axially substituted silicon (IV) phthalocyanine (Br-ID-SiPc) is developed to improve PDT efficiency by enhancing the subcellular localization of photosensitizers. Benefiting from the targeting capability of bromopropylate imidazoliumyl, Br-ID-SiPc can selectively accumulate in mitochondria after cellular uptake, this process could be tracked by two-photon imaging. Br-ID-SiPc effectively damaged the circular plasmid DNA of mitochondria and induced HO-8910 cells apoptosis. Our results indicate that Br-ID-SiPc is a potential photosensitizer which can be used as a mitochondria-targeting and two-photon fluorescent imaging molecule for PDT of cancers.

## Introduction

Photodynamic therapy (PDT) is a noninvasive, negligible drug resistance, spatiotemporally controlled method for the therapy of cancers and noncancer diseases (Uruma et al., 2019). PDT involves a photochemical/photophysical process in which the photosensitizers (PSs) can be activated by a specific wavelength of light to generate reactive oxygen species (ROS) for killing the cancer cells (Chilakamarthi and Giribabu, 2017). However, the very short lifetime (<40 ns) and limited diffusion radius (<20 nm) of ROS generated by the PSs greatly limit the scope of photodynamic action and reduce the intrinsic therapeutic efficacy of PSs (Zhang et al., 2015; Wang et al., 2018). In order to enhance the PDT efficacy, many organelle-targeting PSs have been developed and applied to PDT (Kulsi and Song, 2016). Phthalocyanines (Pcs) are known as promising Type II PSs with good photostability and synthetic tunability (Torre and Tomás, 2002). Several Pcs targeting the organelles such as mitochondria (Dummin et al., 1997; Zhao et al., 2011; Ge et al., 2013; Zhao et al., 2019), lysosomes (Rodriguez et al., 2009) and lipid droplets (Rijcken et al., 2007) have been reported in recent years.

Mitochondria are considered one of the most important organelles that is essential to the energy production, electron transport, calcium mentalism, ROS production and immunity regulation (Liew et al., 2021). Recent studies provide that mitochondrial metabolism is essential in tumorigenesis and plays a multi-functional role in tumor progression (Fogal, 2010; Weinberg et al., 2010). Targeting mitochondria of cancer cells provides the opportunities for the treatment of cancer (Weinberg and Chandel, 2015). Recently, a variety of mitochondrial-targeted molecules have been developed for cancer treatment. It has been reported that mitochondrial-targeted molecules should be both positively charged and lipophilic, which can make use of the mitochondrial membrane potential to selectively accumulate within the organelles (González et al., 2018). Delocalized lipophilic cations (DLCs) show high intrinsic affinity to mitochondria due to the delocalized positive charge and lipophilicity (Murphy, 2008). The negative potential of mitochondrial inner membrane (IMM) (−180 mV) and plasma membrane (−60 mV) can lead DLCs to accumulate 10,000-fold and 10-fold within the mitochondria and the cytoplasm, respectively (Yousif et al., 2009). Targeting molecules to mitochondria based on the potential of mitochondrial membrane is 3- to 5-fold lower than that of the plasma membrane. The negative potential of mitochondrial membrane gives the positively charged molecules an opportunity to enter into the mitochondria, but it is still difficult for many molecules to get into the mitochondrial matrix efficiently cross two different membranes (the outer membrane and the inner membrane). Lipophilic molecules with excellent membrane-permeability are able to easily traverse the mitochondrial membranes by hydrophobic interactions (Ketterer et al., 1971). DLCs such as triphenylphosphonium (TPP) (Zielonka et al., 2017), dequalinium (D'Souza et al., 2008), rhodamine 123 (Baracca et al., 2003) and (E)-4-(1H-indol-3-ylvinyl)-N-methylpyridinium iodide (F16) (Fantin et al., 2002; Fantin and Leder, 2004) have been demonstrated with mitochondrial targeting ability. Many mitochondria-targeting antioxidants and anticancer drugs using DLCs as ligands were developed (Propper et al., 1999; Jones et al., 2005; Kurtoglu and Lampidis, 2009; Qian et al., 2019). Some Pcs with DLC molecules such as cationic trimethylammonio substituted zinc (II) phthalocyanine (Dummin et al., 1997), rhodamine B axially substituted silicon (IV) phthalocyanines (Zhao et al., 2011), cationic 1-(3-methyl) imidazoliumyl ethyloxy substituted zinc (II) phthalocyanine (Ge et al., 2013), and gefitinib and alkylated cationic triphenylphosphonium substituted silicon (IV) phthalocyanine (Zhao et al., 2019) have been demonstrated to target mitochondria.

Imidazole ring is part of the bioactive structure of many drugs such as histidine and the related hormone histamine, as well as many other significant biomolecules. Imidazole derivatives also exhibit antibacterial and antitumor activity (Bellina et al., 2006; Luca, 2006; Zhang et al., 2014). Moreover, 3-bromopropylate imidazoliumyl substituent has both lipophilic and cations characteristics. Based on these facts, we introduced a novel cationic bromopropylate imidazoliumyl ligand to the axial positions of silicon (IV) phthalocyanine to obtain di-(1-(2-hydroxyethyl)-3-(3-bromopropyl) imidazolium) axially substituted silicon (IV) phthalocyanine (Br-ID-SiPc). We demonstrated that Br-ID-SiPc exhibited the ability of targeting mitochondria through two-photon imaging and possessed excellent PDT phototoxicity against human ovarian cancer (HO-8910) cells.

## Experimental Section

### Materials

1,3-Dibromopropane, 1-(2-hydroxyethyl) imidazole and 1,3-diphenylisobenzofuran (DPBF) were purchased from Energy Chemical Company (Shanghai, China). Penicillin−streptomycin solution, silicon phthalocyanine dichloride (SiPcCl_2_) and 10-anthracenediyl-bis(methylene)dimalonic acid (ABDA) were purchased from Sigma-Aldrich. [5-(And-6)-chloromethyl-2′,7′-dichlorodihydrofluorescein diacetate acetyl ester] (CM-H2DCFDA) and Mito Tracker^TM^ Green FM were purchased from Thermo Fisher Scientifific (Shanghai, China). Dulbecco’s minimum essential media (DMEM)/high glucose and fetal bovine serum (FBS) were obtained from Gibco, United States. Cell counting kit-8 (CCK-8) was purchased from Biological Development Co., Ltd. NanjingKGI. HO-8910 cells were supplied by Shanghai Kefeng biological technology Co. (Shanghai, China).

### Synthesis of 1-(2-Hydroxyethyl)-3-(3-Bromopropyl) Imidazolium Bromide

A mixture of 1-(2-hydroxyethyl) imidazole (0.090 g, 0.8 mmol), 1,3-dibromopropane (0.484 g, 2.4 mmol) and acetonitrile (30 mL) was refluxed for 24 h. After being cooled to room temperature and filtrated, a solid was obtained. The precipitate was washed with dichloromethane for several times. Then a white solid was obtained. Yield: 0.3 g (64%). Anal. Calc. For C_8_H_14_Br_2_N_2_O (%): C 30.60, H 4.49, N 8.92; Found: C 30.59, H 4.50, N 8.91. IR ν/cm^−1^: 3285, 3068, 1738, 1629, 1564, 1447, 1360, 1157, 1053, 850, 755, 641, and 565; ^1^H NMR (400 MHz, D_2_O): δ = 8.81 (s, 1H), 7.47 (d, 2H), 4.70 (t, 3H), 4.34-4.23 (m, 4H), 3.86-3.83 (m, 2H), and 2.50-2.32 (m, 2H) (Supplementary Figure S2). ^13^C NMR (400 MHz, D_2_O): δ = 135.9 (C3), 122.9 (C5), 122.3 (C4), 59.7 (C1), 51.8 (C2), 46.5 (C6), 31.5 (C8), and 29.6 (C7) (Supplementary Figure S3). MALDI-TOF-MS m/z: [M-Br]^+^ calcd for C_8_H_14_N_2_OBr^+^ 234.12; found 234.553.

### Synthesis of di-[1-(2-Hydroxyethyl)-3-(3-Bromopropyl) Imidazolium] Axially Substituted Silicon Phthalocyanine

SiPcCl_2_ (0.2 g, 0.32 mmol), Br-ID (0.3 g, 0.98 mmol), K_2_CO_3_ (0.09 g, 0.65 mmol) and toluene (30 mL) were refluxed for 48 h. Methanol was added after the mixture being cooled to room temperature. The mixture was filtrated and the filtrate was concentrated. A solid was obtained and further purified twice on alumina chromatographic column using methanol as eluent. A blue solid was obtained. Yield: 0.15 g (46%). Anal. Calc. For C_48_H_42_Br_2_N_12_O_2_SiCl_2_: C 53.49, H 3.93, N 15.60; Found: C 53.48, H 3.91, N 15.59. IR ν/cm^−1^: 3399, 1651, 1518, 1431, 1167, 1110, 1075, 912, 744, 641, and 570. ^1^H NMR (400 MHz, D_2_O): δ = 9.69–9.67 (m, 8H), 8.51–8.49 (m, 8H), 7.49 (s, 2H), 7.48 (d, 4H), 4.25–4.22 (t, 12H), 3.85–3.83 (t, 4H), 2.47–2.45 (m, 4H) (Supplementary Figure S6). ^13^C NMR (400 MHz, CDCl_3_): δ = 150.1 (C1), 135.4 (C2), 131.6 (C4, C7), 124.1 (C3, C8, C9), 64.4 (C5), 53.5 (C6, C10), and 29.8 (C11, C12) (Supplementary Figure S7). MALDI-TOF-MS m/z: [M + Na]^+^ calcd for C_48_H_42_Br_2_N_12_O_2_SiCl_2_Na^+^ 1097.11; found 1097.207.

### Photochemical Parameters

The fluorescence lifetime (*τ*_*s*_), fluorescence quantum yield (*Φ*_*F*_) and singlet oxygen quantum yield (*Φ*_*Δ*_) of Br-ID-SiPc were determined and calculated using experimental methods provided in Supplementary materials.

### Cell Culture

HO-8910 cells were cultured in DMEM containing 10% (v/v) high glucose and FBS and 1% (v/v) penicillin-streptomycin at 37°C with 5% CO_2_.

### Intracellular Uptake

HO-8910 cells were plated onto a 12-well plate and incubated at 37°C in 5% CO_2_ for 24 h. Br-ID-SiPc (1.0 × 10^–5^ mol/L, 10 µL) was then added. HO-8910 cells were incubated for 4 h and observed using confocal laser scanning microscopy (CLSM).

### Subcellular Localization

HO-8910 cells were plated into 20 mm confocal dishes for 24 h at 37°C in 5% CO_2_ and subsequently treated with Br-ID-SiPc (1.0 × 10^–5^ mol/L, 10 µL) for 6 h. The medium was removed and the cells were washed with PBS. The cells were fixed in 4% formaldehyde for 15 min at room temperature and then washed with PBS. 0.2% Saponin was added and incubated at room temperature for 10 min. Cells were then stained with Mito Tracker^TM^ Green FM (50 nM, 1 mL), a mitochondria-specific probe at room temperature for 10 min in the dark. The emitted fluorescent signals of Br-ID-SiPc and Mito Tracker Tracker^TM^ Green FM were observed using CLSM. Mito Tracker^TM^ Green FM and Br-ID-SiPc were excited at 488 nm by one-photonic laser and 730 nm by two-photon laser, respectively. Their fluorescence signals were monitored at 500–550 nm and 650–750 nm.

### Reactive Oxygen Species Generation Ability

HO-8910 cells were plated in 20 mm glass bottom dishes and cultured at 37°C in 5% CO_2_ for 24 h. The cells were treated with Br-ID-SiPc (1.0 × 10^–5^ mol/L, 10 µL) for 12 h. The experimental group was irradiated with 671 nm laser at 32 mW/cm^2^ for 5 min and the control group was not irradiated by laser. Then both groups were treated with CM-H2DCFDA (5 μM) and incubated for 20 min and observed using CLSM.

### Cell Counting Kit-8 Assay

CCK-8 assay was used to evaluate the dark cytotoxicity and phototoxicity of Br-ID-SiPc against HO-8910 cells. HO-8910 cells with a density of 8 ×10^3^ cells per well were plated and cultured for 24 h. After finishing the culturing, the cells were treated with different concentrations of Br-ID-SiPc (0 μM, 1 μM, 2 μM, 3 μM, 4 μM, and 5 μM) and further cultured for 6 h. Then the cells were irradiated with 671 nm laser (100 mW/cm^2^) for 10 min and cultured for 4 h. Finally, CCK-8 reagent (10 μL) was added into each well and the cells were incubated for 2 h. The OD value at 450 nm was detected by a multifunction microplate reader.

### Flow Cytometry Analysis

Cell apoptosis was analyzed by flow cytometry using Annexin V-FITC/Propidium Iodide staining. HO-8910 cells with a density of 1×10^5^ cells per well were plated in 24-well plates and cultured for 24 h. The cells were treated with Br-ID-SiPc (1.0 × 10^–5^ mol/L, 10 µL) in fresh DMEM. In the control group, the culture medium was replaced by fresh DMEM and the cells were cultured for 6 h. The cells in illumination groups were irradiated by 671 nm laser (100 mW/cm^2^) for 5 min and washed with cold PBS for three times. The cells were then stained with both annexin V-FITC (5 μL) and Propidium Iodide (5 μL) for 15 min in the dark. The apoptosis of cells was measured by flow cytometer.

### DNA Damage

DNA damage by Br-ID-SiPc was measured by agarose gel electrophoresis using covalently closed circular plasmid DNA (cccDNA) as a model. The cccDNAs (100 ng/μL, 1 μL) was treated with Br-ID-SiPc (1.0 × 10^–5^ mol/L, 10 μL) in the Tris-HCl buffer (50 mM, pH 7.0) for 10 min. Then the DNA solutions were irradiated under light (671 nm, 17.5 mW/cm^2^) for 0, 3, 6, 9, 12, 15, and 18 min, respectively. At the same time, control groups were set up to examine the effects of only light or Br-ID-SiPc on DNA damage. After irradiation, The DNA solutions were loaded on a 0.8% agarose gel with ethidium bromide dye in TAE (Tris-acetic acid-EDTA) buffer. Electrophoresis was carried out at 100 V for 60 min and the DNA bands were visualized under a UV transilluminator and photographed.

## Results and Discussion

### Synthesis and Characterization

**FIGURE 1:**
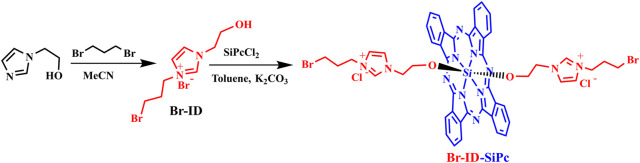
The synthetic route of Br-ID-SiPc.

The synthetic route for Br-ID-SiPc was shown in Figure 1. The precursor 1-(2-hydroxyethyl)-3-(3-bromopropyl) imidazolium bromide (Br-ID) was synthesized *via* nucleophilic substitution of 1-(2-hydroxyethyl) imidazole with 1,3-dibromopropane. Br-ID further reacted with SiPcCl_2_ to obtain Br-ID-SiPc with a yield of 46%. Characterization of Br-ID and Br-ID-SiPc were carried out by elemental analysis, IR, ^1^H NMR,^1^C NMR and MALDL-TOF mass spectroscopic methods (Supplementary Figures S1–S8).

### Photophysical and Photochemical Properties

**FIGURE 2:**
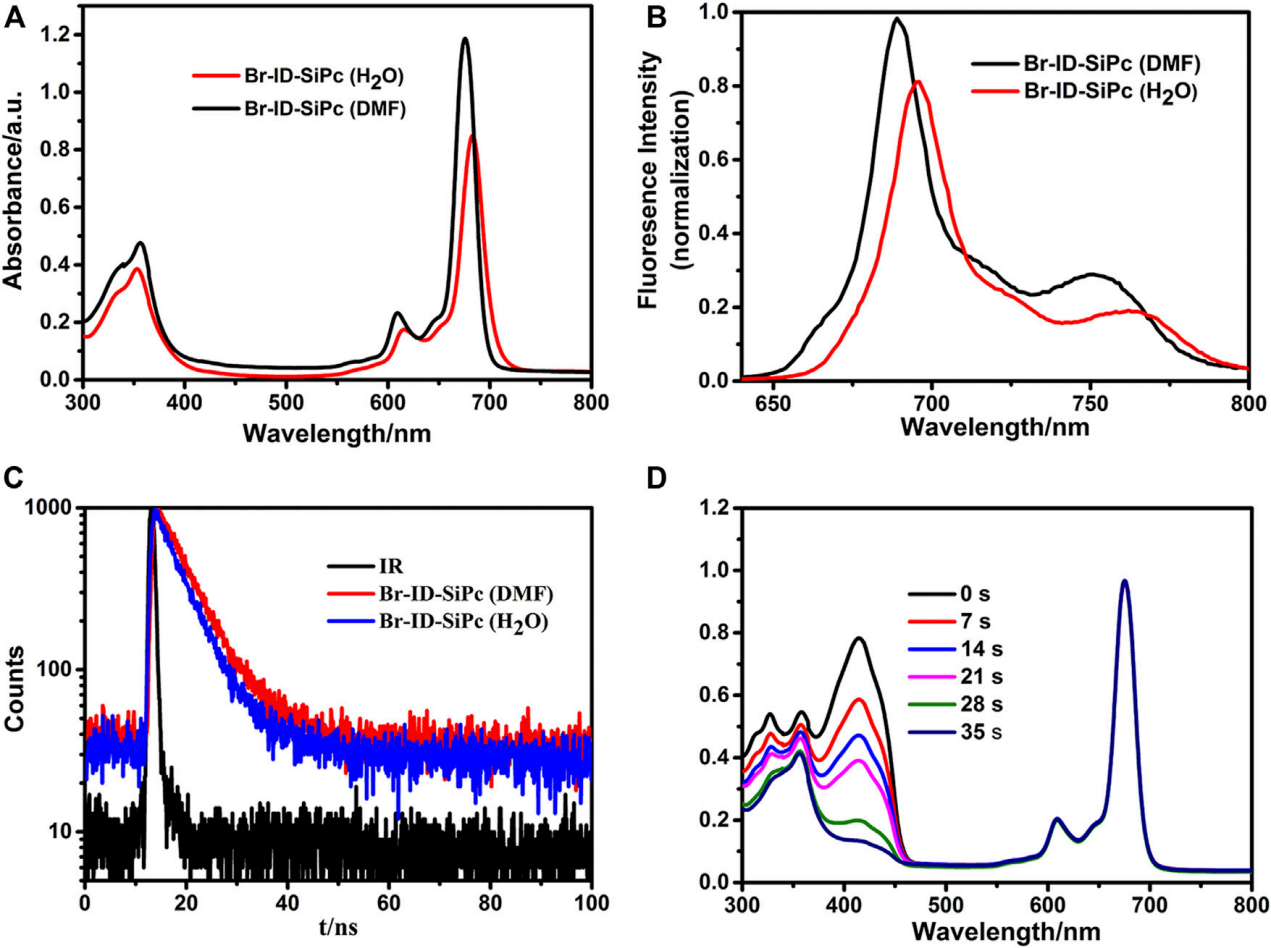
UV/Vis spectra **(A)**, fluorescence spectra **(B)** and fluorescence decay curves **(C)** of Br-ID-SiPc in DMF and H_2_O; The spectrum changes during the determination of singlet oxygen quantum yield of Br-ID-SiPc in DMF **(D)**. (**(A)**: *C*_Br-ID-SiPc_ = 10 μM; **(B)** *C*_Br-ID-SiPc_ = 1.0 μM, λ_ex(Br-ID-SiPc)_ = 610 nm; **(C)**: *C*_Br-ID-SiPc_ = 10 μM, λ_ex(Br-ID-SiPc)_ = 405 nm; **(D)**: *C*_Br-ID-SiPc_ = 8 μM).

UV/Vis spectra of Br-ID-SiPc in *N*, *N*-dimethylformamide (DMF) and H_2_O were shown in Figure 2A. Br-ID-SiPc exhibited characteristic spectra of phthalocyanine with a B band at 356 nm and a Q band at 676 nm in DMF solution, and a B band at 353 nm and a Q band at 683 nm in H_2_O. A strong typical non-aggregated sharp Q-band was observed in different concentrations of Br-ID-SiPc (Supplementary Figure S9), suggesting that Br-ID-SiPc mainly existed in the state of monomer in DMF or H_2_O. The steric hindrance effect of bromopropylate imidazoliumyl substituents at the axial positions probably limited phthalocyanine aggregation in DMF and H_2_O. When the solvent was changed from DMF to H_2_O, a red shift from 676 to 683 nm and a decrease in absorption intensity were observed, which may be contributed to the change of intermolecular solute-solvent interaction forces in different solutions (Yüksel et al., 2003) or the aggregation behavior of Br-ID-SiPc in H_2_O.

Fluorescence spectra of Br-ID-SiPc in *N*, *N*-dimethylformamide (DMF) and H_2_O were shown in Figure 2B. When the solvent was changed from DMF to H_2_O, a red shift from 689 to 696 nm and a decrease in fluorescence intensity were observed. The fluorescence quantum yield (*Φ*_F_) of Br-ID-SiPc in DMF and in H_2_O were found to be 0.39 and 0.32, respectively. The fluorescence lifetime of Br-ID-SiPc was found to be 6.06 ns in DMF and 5.36 ns in H_2_O (Figure 2C). Singlet oxygen production ability of Br-ID-SiPc was assessed using DPBF in DMF (Figure 2D) or ABDA in aqueous solution (Supplementary Figure S10) as the scavenger. The singlet oxygen quantum yield (*Φ*_Δ_) of Br-ID-SiPc was found to be 0.377 in DMF and 0.121 in H_2_O. These results indicated that Br-ID-SiPc was a water-soluble photosensitizer and exhibited excellent photochemical properties for potential PDT application.

### Subcellular Localization of Br-ID-SiPc in HO-8910 Cells

**FIGURE 3:**
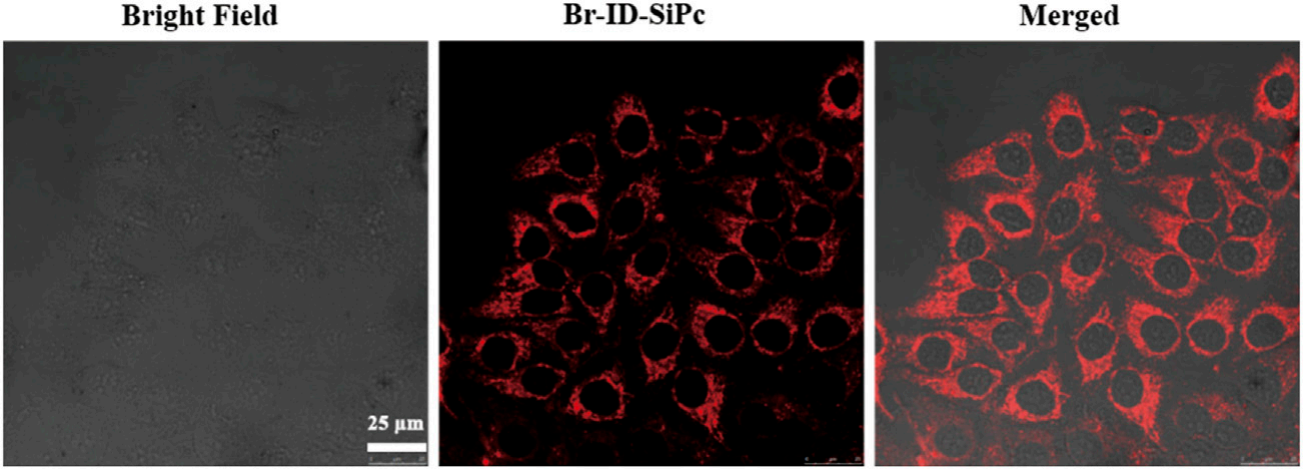
Confocal microscopy fluorescence images of HO-8910 cells after being incubation with Br-ID-SiPc for 4 h (λ_ex_ = 860 nm, λ_em_ = 650–750 nm).

HO-8910 (human ovarian cancer cell line) cell line was employed as a model cell line to explore the subcellular localization and PDT potential of Br-ID-SiPc. The two-photon images of Br-ID-SiPc were shown in Supplementary Figure S11. The strong red fluorescence of Br-ID-SiPc in two-photon laser allowed us to visualize the intracellular uptake and subcellular localization of Br-ID-SiPc in HO-8910 cells. After incubation with Br-ID-SiPc in HO-8910 cells for 4 h, strong red dotted fluorescence was observed in the cytoplasm by two-photon confocal fluorescence microscopy (Figure 3).

**FIGURE 4:**
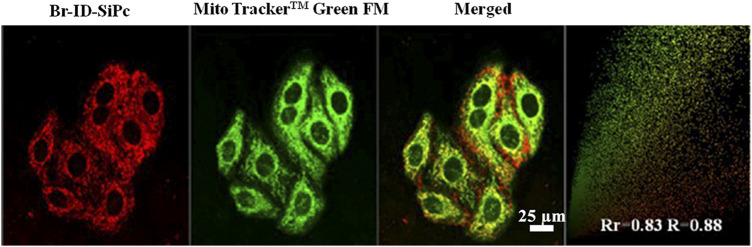
Confocal microscopy fluorescence images of Br-ID-SiPc (red, λ_ex_ = 860 nm, λ_em_ = 650–750 nm) and MitoTracker™ Green FM (green, λ_ex_ = 488 nm, λ_em_ = 500–550 nm) in HO-8910 cells.

After being uptaken by HO-8910 cells, the Br-ID-SiPc was not evenly distributed in the cytoplasm and exhibited a dotted pattern in cells, indicating that Br-ID-SiPc may be localized in some specific functional regions of cells. The lipophilic positively charged moiety of bromopropylate imidazoliumyl substituents in the Br-ID-SiPc molecules allowed it to interact with membrane system of mitochondria which possessed the negative potential and lipophilic character, therefore, the Br-ID-SiPc molecules could be located in the mitochondria after being uptaken into the HO-8910 cells. A experiment was performed. The Br-ID-SiPc molecules were incubated with HO-8910 cells for 12 h and then costained with Mito Tracker^TM^ Green FM, a mitochondrial fluorescence probe, for 30 min. As shown in Figure 4, the red fluorescence from Br-ID-SiPc exhibited overlap with green fluorescence from Mito Tracker^TM^ Green FM. The Pearson’s correlation coefficient (PCC) and Mander’s overlap coefficient (MOC) were about 0.83 and 0.88, respectively. The results suggested that Br-ID-SiPc mainly localized in mitochondria of HO-8910 cells.

### *In Vitro* Photodynamic Therapy Efficacy of Br-ID-SiPc Against HO-8910 Cells

**FIGURE 5:**
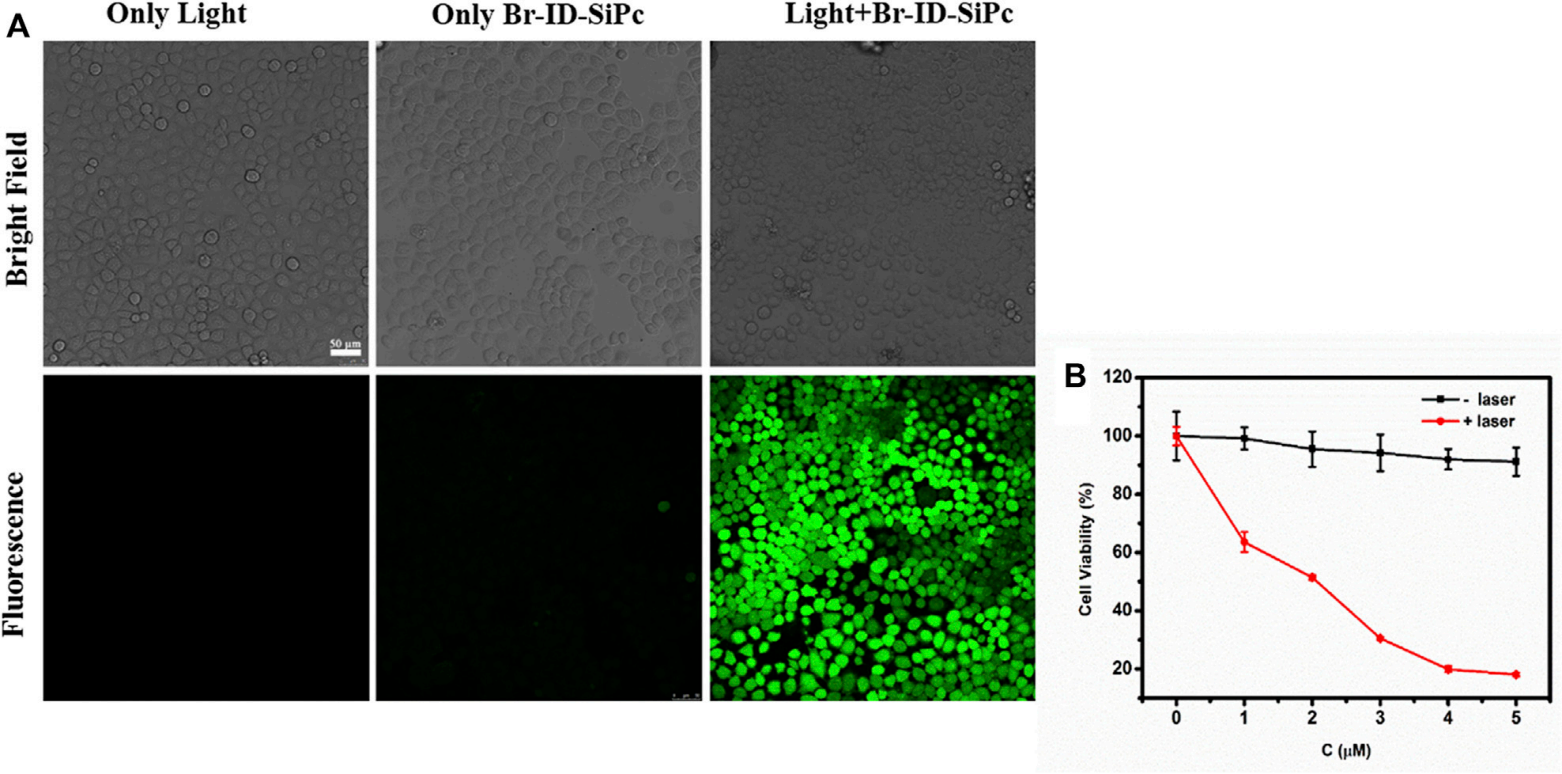
ROS generation in HO-8910 cells was detected by confocal microscopy fluorescence imaging using CM-H2DCFDA fluorescent probe (*C*_CM-H2DCFDA_ = 10 μM; λ_ex_ = 488 nm, λ_em_ = 490–590 nm) **(A)**; Viability of HO-8910 cell treated by Br-ID-SiPc in the absence or presence of light **(B)**.

No obviously photodegradation was observed for Br-ID-SiPc upon irradiation at 671 nm for 200 min, therefore, Br-ID-SiPc exhibited excellent photostability (Supplementary Figure S12). No obvious photothermal effect was also observed for Br-ID-SiPc under laser irradiation (Supplementary Figure S13). Therefore, the *in vitro* photodynamic therapy efficacy of Br-ID-SiPc against HO-8910 cells was evaluated. ROS generation ability is an important indicator for a photosensitizer to evaluate its PDT efficiency. The intracellular ROS generation by Br-ID-SiPc in HO-8910 cells was examined by fluorescence microscopy using the CM-H2DCFDA as a fluorescent probe. As shown in Figure 5A, no fluorescence was observed in the treatment of Br-ID-SiPc without light, but strong green fluorescence was observed in HO-8910 cells when cells were simultaneously treated with Br-ID-SiPc and light, indicating that Br-ID-SiPc excited by light exhibited strong ROS generation ability. The cytotoxicity of Br-ID-SiPc in HO-8910 cells was evaluated by CCK-8 assay. Figure 5B showed that there was almost no cytotoxicity for Br-ID-SiPc to HO-8910 cells without light. Upon excited by light, Br-ID-SiPc demonstrated a strong ability to kill HO-8910 cells. Cell viability decreased to 20% after irradiation with 671 nm laser at 100 mW/cm^2^ for 10 min and the IC_50_ value was found to be 2.0 μM against HO-8910 cells.

**FIGURE 6:**
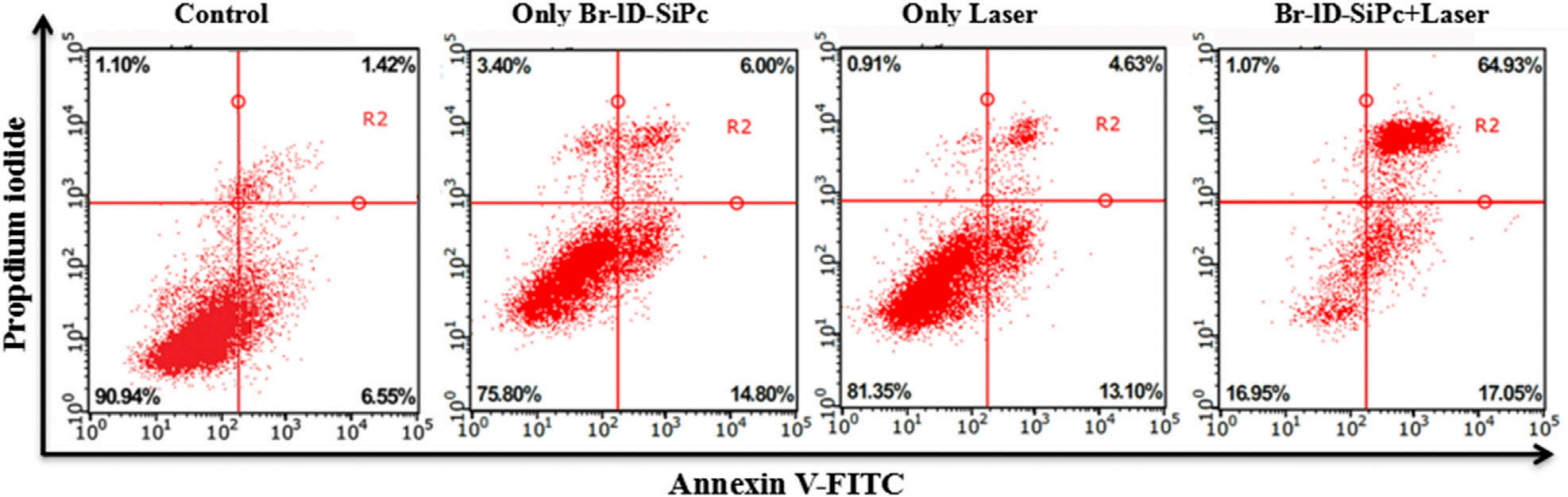
Assessment of apoptosis in HO-8910 cells after treatments with Br-ID-SiPc or/and laser.

Cell apoptosis was detected using Annexin V-FITC/PI apoptosis detection Kit by flow cytometry. The early apoptotic and late apoptotic cells obviously increased after HO-8910 cells were treated by Br-ID-SiPc under the laser light (Figure 6). However, the cells exhibited a slightly increased percentage of both the early apoptotic cells and late apoptotic cells compared with the control group when cells were treated with Br-ID-SiPc or light irradiation alone.

### DNA Cleavage Studies

**FIGURE 7:**
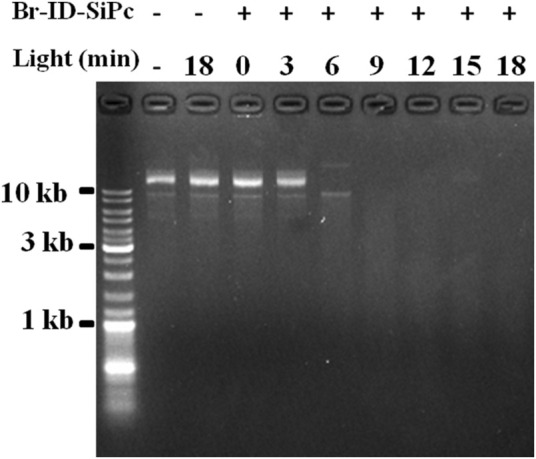
Agarose gel electrophoresis analysis of covalently closed circular plasmid DNA treated with Br-ID-SiPc (1.0 μM) under irradiation.

A circular DNA, called mitochondrial DNA (mtDNA), is located in the mitochondrion. mtDNA encodes several proteins that are needed to function in the pathways for producing energy, and is important for normal function of mitochondria. Mitochondrial DNA damage can trigger mitochondrial dysfunction and induce apoptosis (Kim et al., 2010). ROS is an important factor to damage the mitochondrial DNA. Br-ID-SiPc was found to locate in mitochondria in HO-8910 cells after being uptaken and had a strong ability of ROS generation under the light. Whether could Br-ID-SiPc damage circular DNA? In order to answer the question, tests were performed in the tubes. Purified circular plasmids and Br-ID-SiPc were added into several tubes. Meanwhile, several control groups were set up. After irradiation, DNA damage was examined by agarose gel electrogenesis. Figure 7 showed that circular DNA was damaged after irradiation for 6 min and completely degraded after irradiation for 9 min. The mitochondrial DNA could be damaged by Br-ID-SiPc after PDT which was likely to be a key factor to induce the death of HO-8910 cells.

## Conculsion

A water-soluble cationic bromopropylate imidazoliumyl axially substituted silicon (IV) phthalocyanine (Br-ID-SiPc) was synthesized and characterized. The intracellular uptake and subcellular localization of mitochondria for Br-ID-SiPc in HO-8910 cells were visualized by two-photon fluorescence imaging. Br-ID-SiPc could effectively produce ROS and exhibit outstanding phototoxicity effect on HO-8910 cells leading to the cell death through destroying the DNA of the mitochondria and inducing cell apoptosis. These results obviously stressed the potential application of Br-ID-SiPc as a mitochondrion-targeted photosensitizer candidate for two-photon imaging-guided therapy of cancers.

## Data Availability

The original contributions presented in the study are included in the article/Supplementary Material, further inquiries can be directed to the corresponding authors.
