# Supplementary material for: Bromopropylate Imidazoliumyl Substituted Silicon Phthalocyanine for Mitochondria-Targeting, Two-Photon Imaging Guided in Vitro Photodynamic Therapy
Source: Front Pharmacol. 2022 Jul 12;13:921718. doi: 10.3389/fphar.2022.921718 (PMC9315426; doi:10.3389/fphar.2022.921718)
Supplement: Supplementary file 1 [file DataSheet1.pdf]

Supporting Information

Photochemical Parameters.

The fluorescence quantum yield was determined in DMF using Eq. 1 (Fery-Forgues and Lavabre, 1999; Fu et al., 2002)) and unsubstituted ZnPc (Φ_F=0.28) as the standard (Scalise and Durantini, 2005).

$$\Phi_F = \Phi_{F_{std}} \times {F}/{F_{std}} \times {A_{std}}/{A} \qquad \text{(Eq.1)}$$

In Eq. 1, A and A_{std} are the absorbance of the phthalocyanine and the standard, unsubstituted ZnPc (n-ZnPc) at the excitation wavelengths, respectively. F and F_{std} are the integral area of emission curves of the phthalocyanine and n-ZnPc, respectively.

Singlet oxygen quantum yield (Φ_Δ) in DMF was determined using the chemical trapping method (Förster, 1948; Nyket al., 2010) using n-ZnPc (in DMF) as a reference. DPBF was used as a chemical quencher for singlet oxygen. Br-ID-SiPc that contained diphenylisobenzofuran (**DPBF**) in N, N-dimethylformanide (DMF) was prepared in the dark and irradiated at 671 nm, respectively. The Φ_Δ value was obtained by using **n-ZnPc** as the reference:

$$\Phi_\Delta=\Phi_\Delta^{ref}.(k.\ I_a^{ref})/(k^{ref}.\ I_a) \qquad \text{(Eq.2)}$$

Where Φ_Δ^{ref} is the singlet oxygen quantum yield for the reference (0.56 for n-ZnPc in DMF) ; k and k^{ref} are the DPBF photobleaching rate constants in the presence of the sample and the reference, respectively; I_a and I_a^{ref} are the rates of light absorption by the sample and the reference, respectively. Their ratios can be obtained by Eq. 3

$$I_a^{ref}/I_a=(1\text{-}10^{-A_{670}^{ref}})/(1\text{-}10^{-A_{670}}) \qquad \text{(Eq.3)}$$

To avoid chain reactions induced by DPBF in the presence of singlet oxygen , the concentration of DPBF was lowered to~3× 10^{-5} mol.dm^{-3}. The degradation of DBPF at 414 nm was monitored by UV/Vis absorption spectra.

Singlet oxygen quantum yield (Φ_Δ) in H_2O was determined using ABDA as an indicator (Ge et al., 2014). The absorbance decrease of ABDA at 378 nm was recorded for different durations of light irradiation to obtain the decay rate of the photosensitizing process. Using Rose Bengal (RB) as a reference, the singlet oxygen quantum yield of Br-ID-SiPc in H_2O was calculated according to the following formula:

$$\Phi_\Delta=\Phi_{RB}.(k.\ A_{RB})/(k_{RB}.\ A) \qquad \text{(Eq.4)}$$

Where K and K_{RB} are the decomposition rate constants of ABDA by Br-ID-SiPc and RB. A and A_{RB} represent the light absorbed by Br-ID-SiPc and RB, which are determined by integration of the absorption bands in the wavelength range of 300–800 nm. Φ_{RB} is the singlet oxygen quantum yield of RB, which is 0.75 in H_2O.

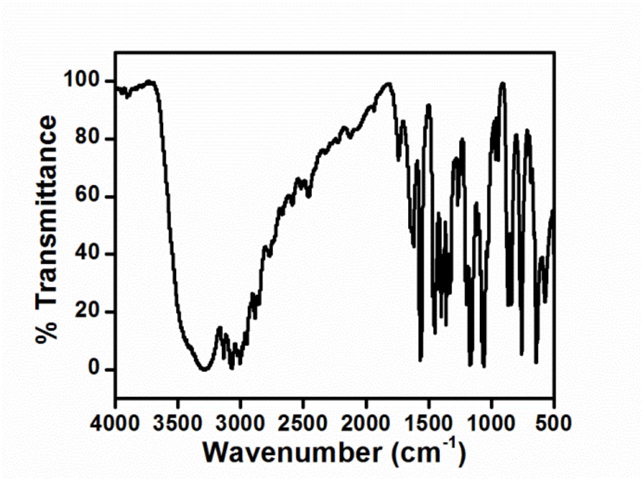

Fig. S1 The FT-IR spectrum of Br-ID

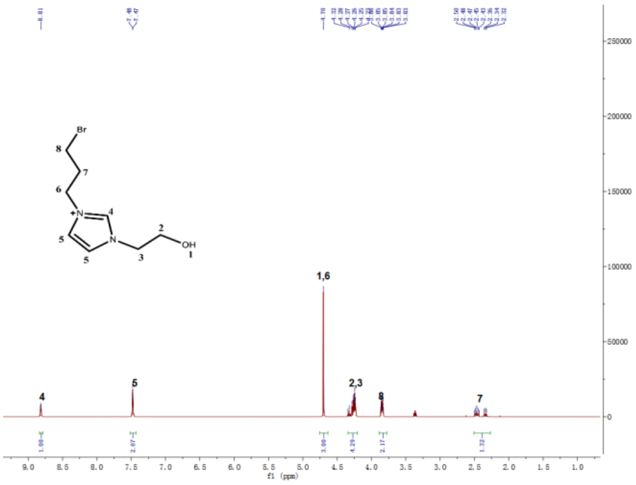

Fig. S2 ^{1}H NMR spectrum of Br-ID (400 MHz, D_2O)

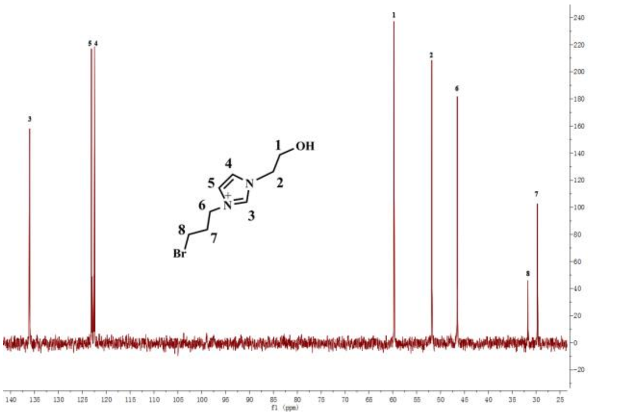

Fig. S3 ^{13}C NMR spectrum of Br-ID (400 MHz, D_2O)

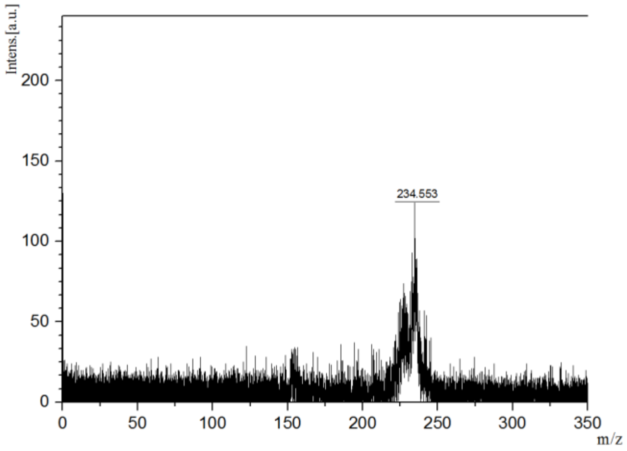

Fig. S4 MALDI-TOF-MS spectrum of Br-ID

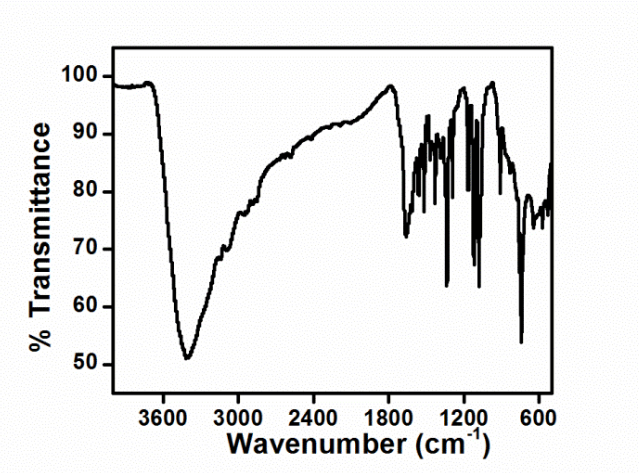

Fig. S5 FT-IR spectrum of Br-ID-SiPc

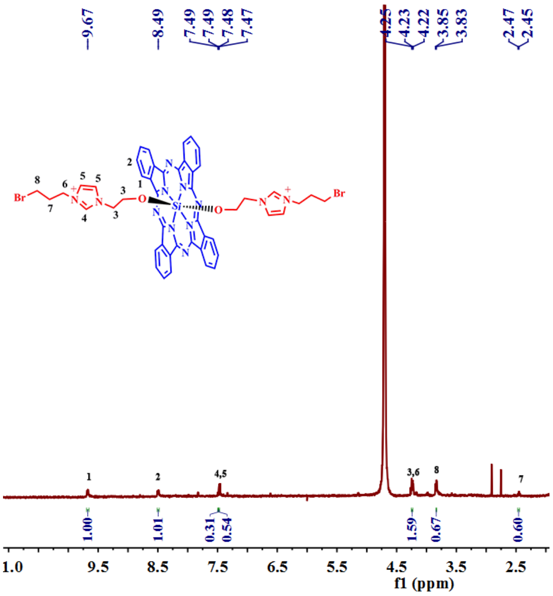

Fig. S6 ^{1}H NMR spectrum of Br-ID-SiPc (400 MHz, DMSO-d_6)

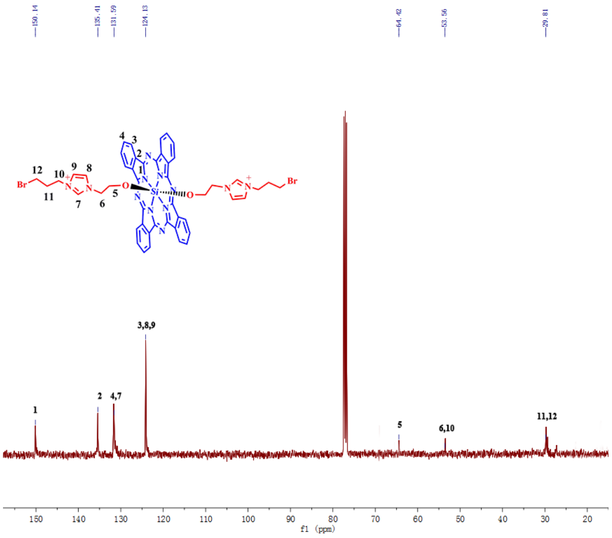

Fig. S7 The ^{1}C NMR spectrum of Br-ID-SiPc (400 MHz, $CDCl_3$)

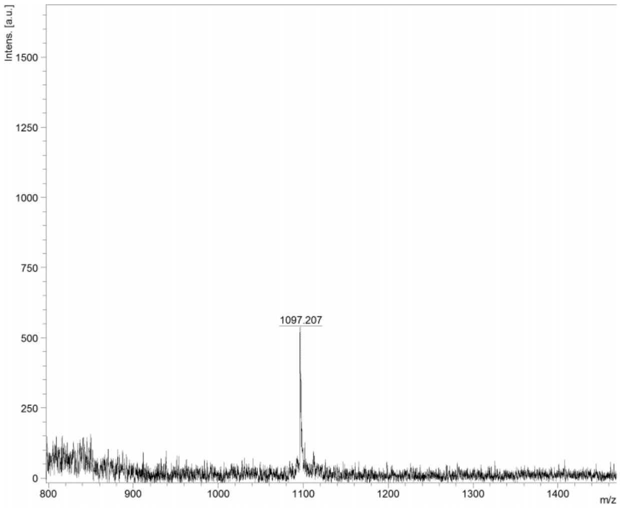

Fig. S8 MALDI-TOF-MS specturm of Br-ID-SiPc

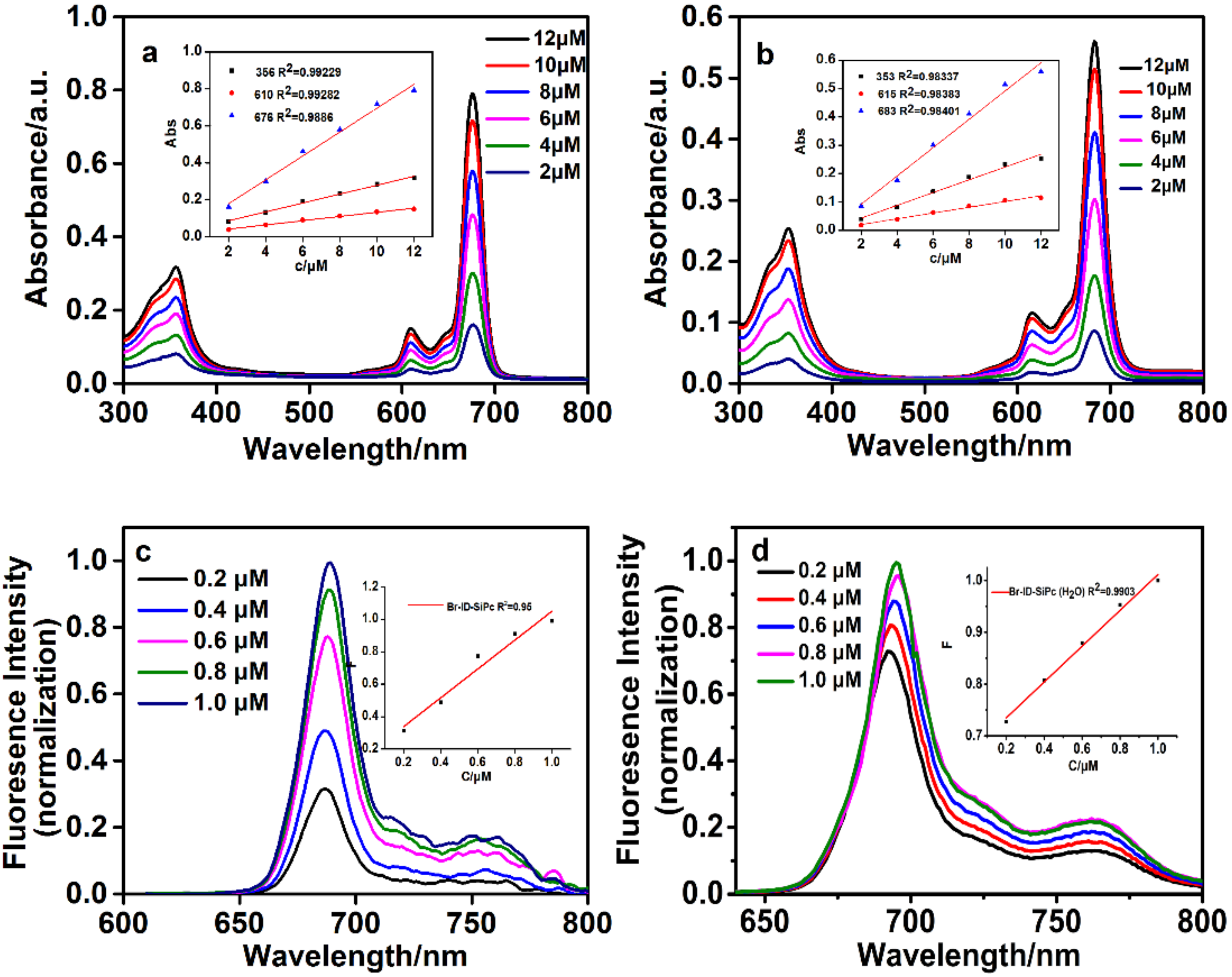

Fig. S9 UV/Vis and fluorescence spectra of Br-ID-SiPc in DMF (a, c) and H_2O (b,d) (λ_{ex}= 610 nm)

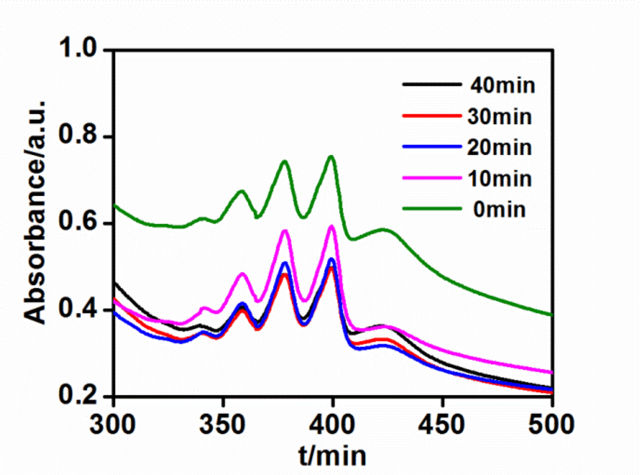

Fig. S10 The spectrum changes during the determination of singlet oxygen quantum yield for Br-ID-SiPc in H_2O. ($C_{Br\text{-}ID\text{-}SiPc}$=1.0×10^{-5} mol/L)

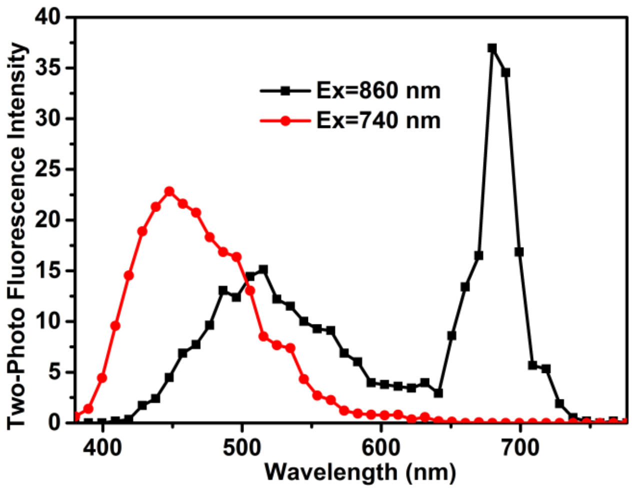

Fig. S11 Two-photon fluorescence spectra of Br-ID-SiPc（λex = 740 nm or 860 nm）

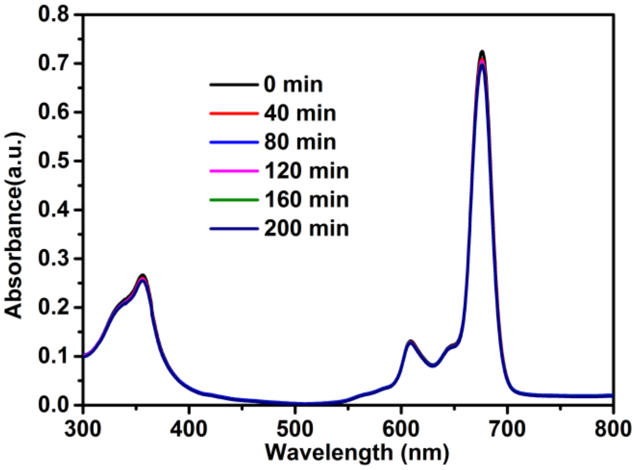

Fig. S12 UV-Vis spectra of Br-ID-SiPc in DMF with different laser irradiation time. (671 nm, 100 mW/cm^2）

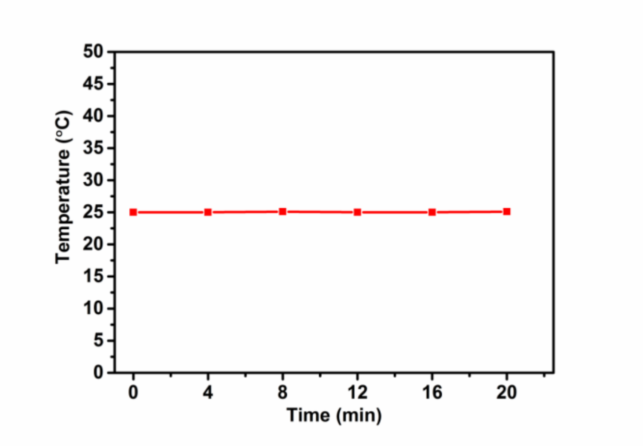

Fig. S13 Temperature curves of Br-ID-SiPc in DMF under 671 nm laser irradiation (100 mW /cm^{-2}).

REFERENCES

Fery-Forgues, S., Lavabre, D. (1999). Are Fluorescence Quantum Yields So Tricky to Measure? A Demonstration Using Familiar Stationery Products. *J. Chem. Educ.* 76, 1260-1264. doi:10.1021/ed076p1260.

Fu, J., Li, X., Ng, D.K.P., Wu, C. (2002). Encapsulation of Phthalocyanines in Biodegradable Poly(sebacic anhydride) Nanoparticles. *Langmuir.* 18, 3843-3847.doi:10.1021/la011764a

Förster, T. (1948). Intermolecular energy transference and fluorescence. *J Ann. Phys.* (2), 55-57.

Ge, J., Lan, M., Zhou, B., Liu, W., Guo, L., Wang, H., et al. (2014). A graphene quantum dot photodynamictherapy agent with high singlet oxygen generation. *Nat. Commun.* 5, 4596-4603. doi:10.1038/ncomms559 6

Nyk, M., Palewska, K., Kepinski, L., Wilk, K. A., Strek, W., Samoc, M. (2010). Fluorescence resonance energy transfer in a non-conjugated system of CdSe quantum dots/zinc-phthalocyanine. *J Lumin.* (130), 2487-2490.doi:10.1016/j.jlumin.2010.08.017

Scalise, I., Durantini, E. N.(2005). Synthesis, properties, and photodynamic inactivation of Escherichia coli using a cationic and a noncharged Zn(II) pyridyloxyphthalocyanine derivatives. *Bioorg. Med. Chem.* 13, 3037-3045.doi:10.1016/j.bmc.2005.01.063
